# Supplementary figures and images for: Glycolytic flux controls retinal progenitor cell differentiation via regulating Wnt signaling
Source: eLife. 2025 Jun 17;13:RP100604. doi: 10.7554/eLife.100604 (PMC12173459; doi:10.7554/eLife.100604)

Figure 6D

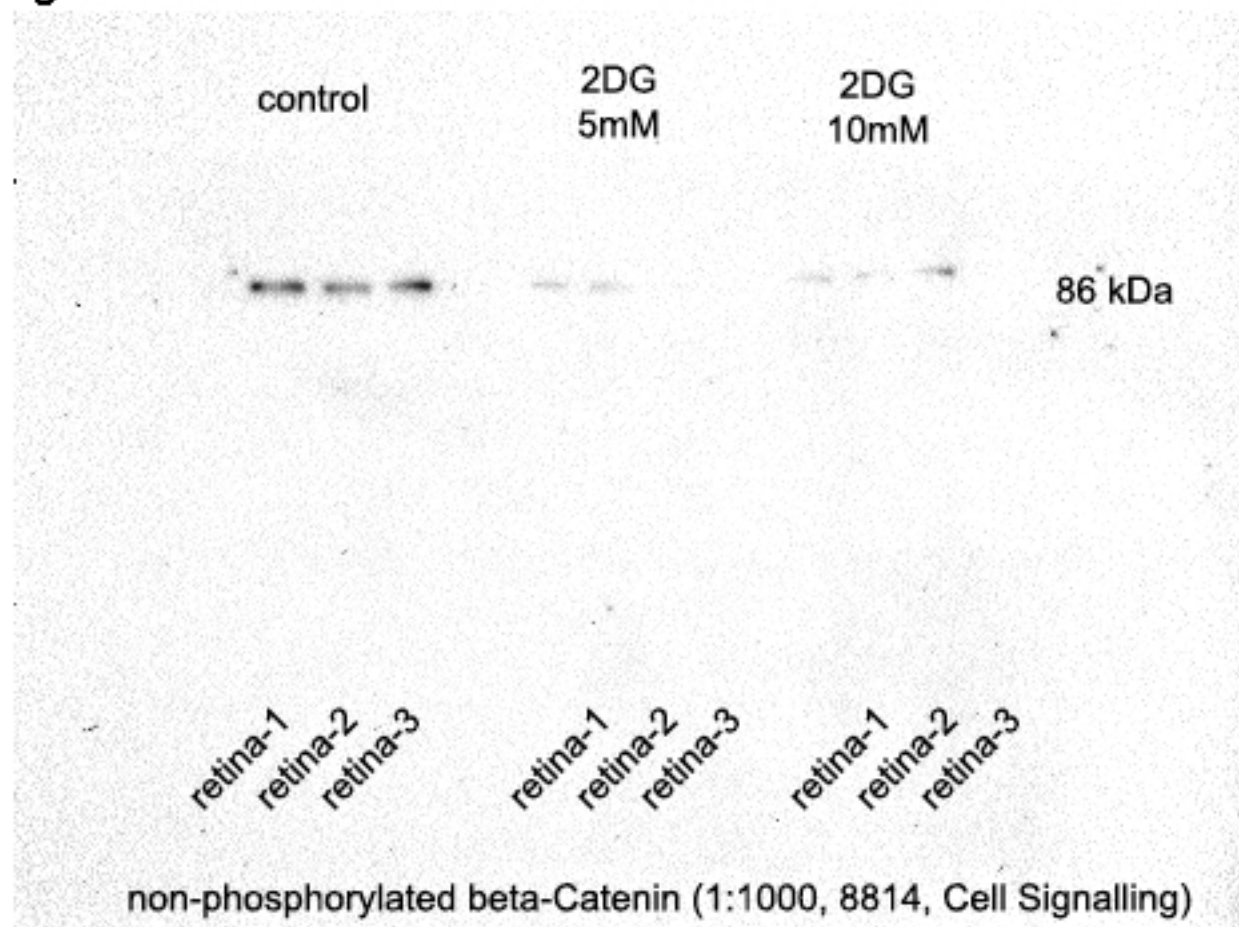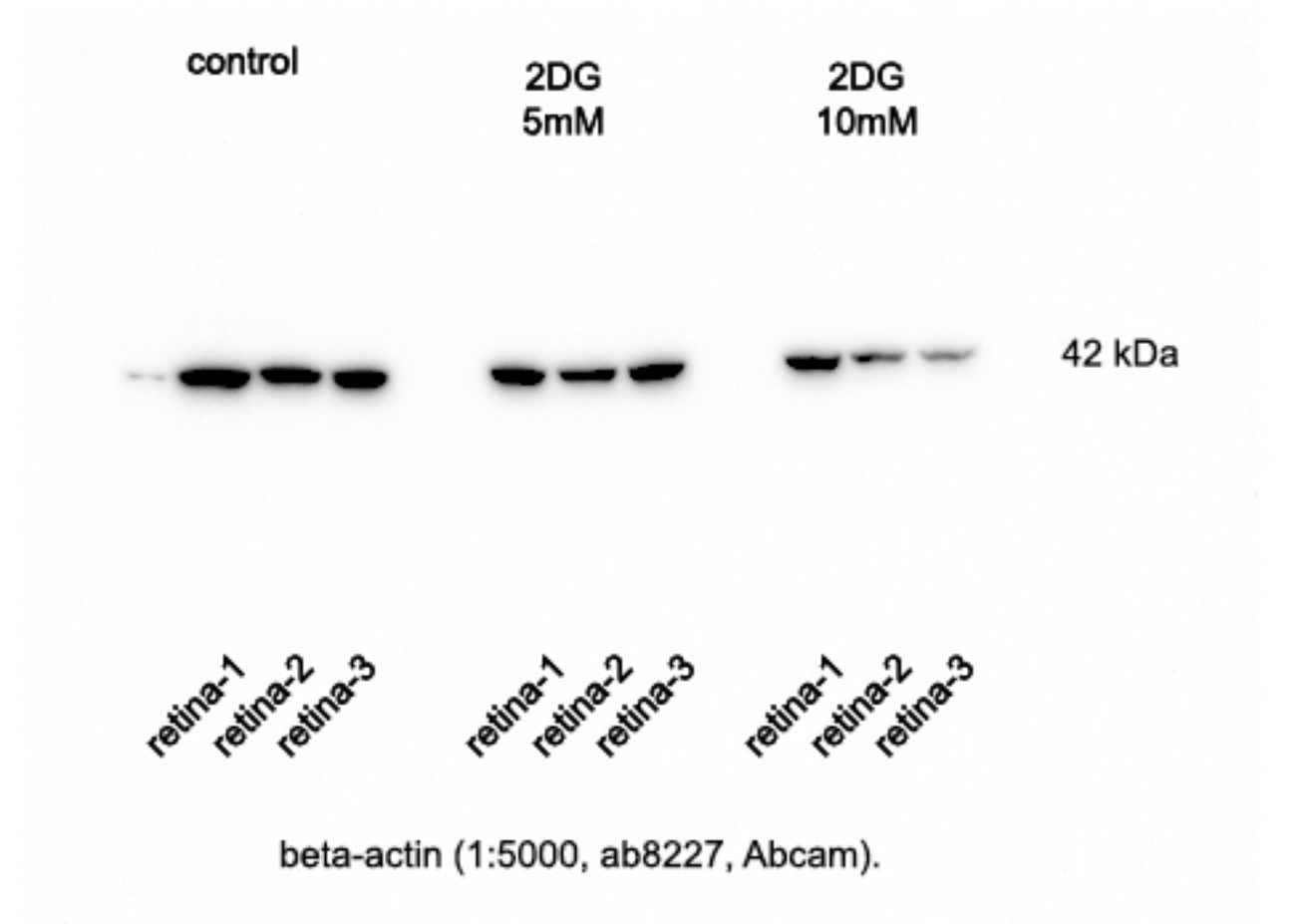

Figure 6E

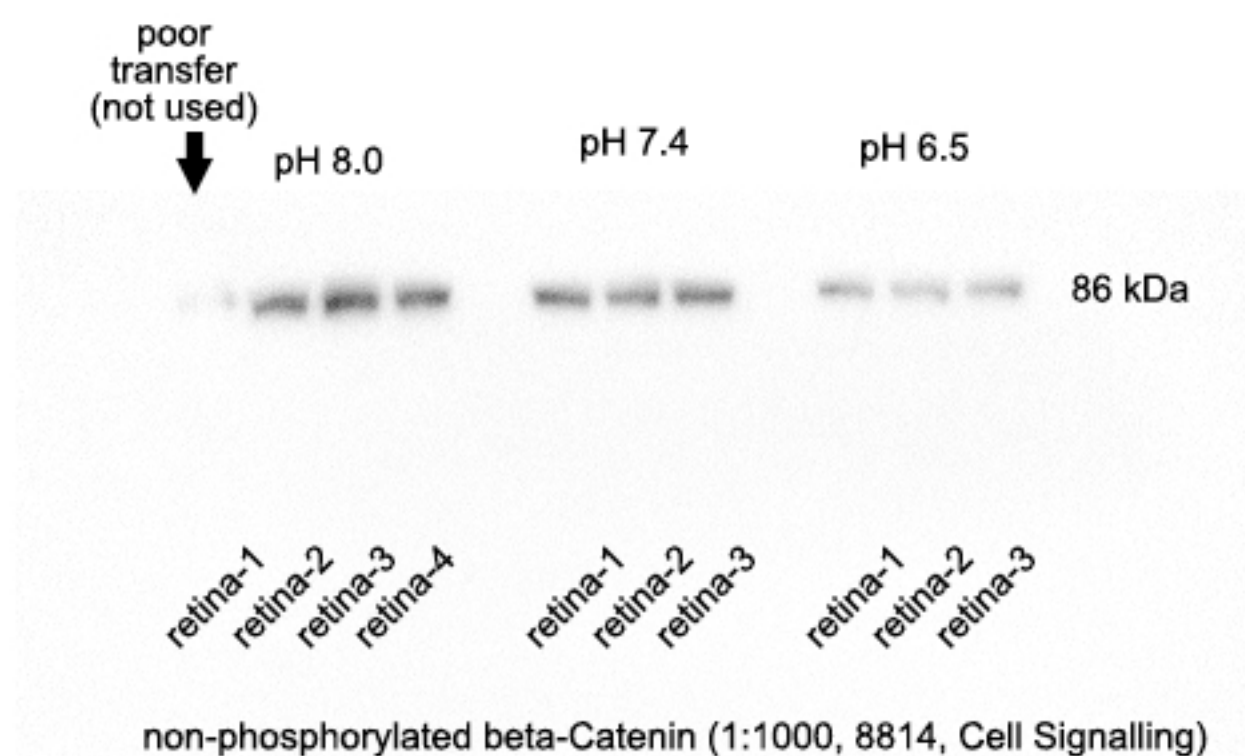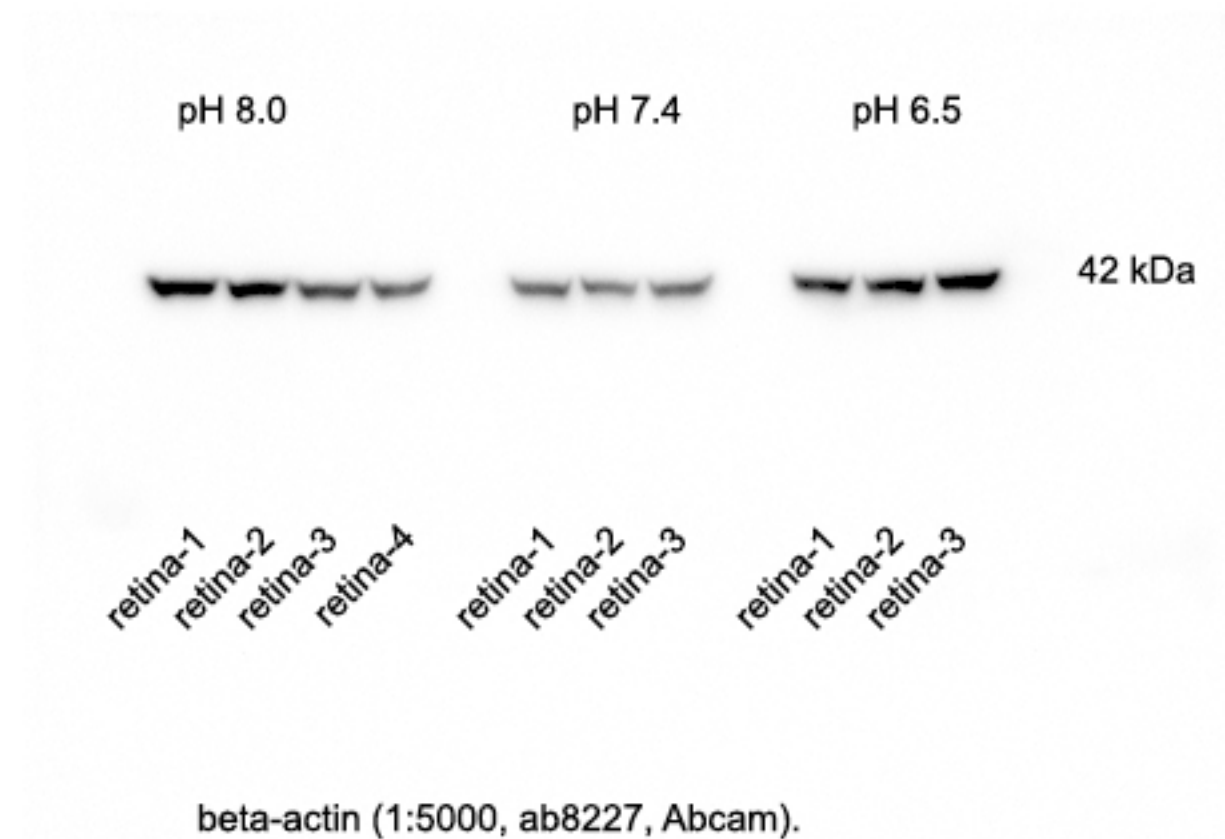

Supplement: Figure 6—source data 1. [file elife-100604-fig6-data1.zip › Figure 6_source data 1/Figure 6_source data 1.pdf]

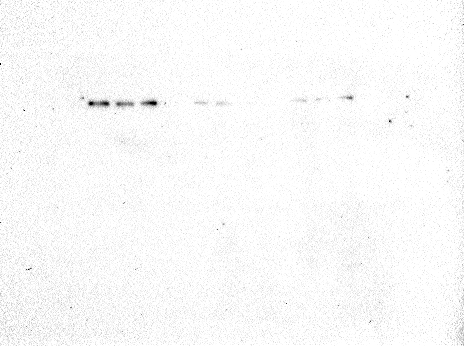

Supplement: Figure 6—source data 2. [file elife-100604-fig6-data2.zip › Fig. 6D (b-catenin).tif]

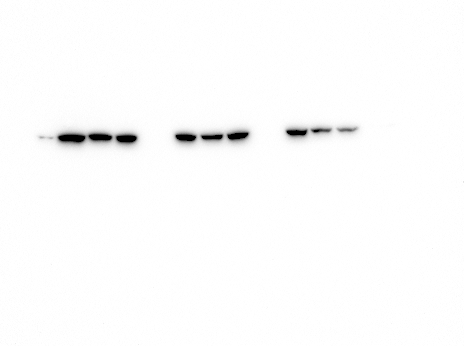

Supplement: Figure 6—source data 2. [file elife-100604-fig6-data2.zip › Fig. 6D (b-actin).tif]

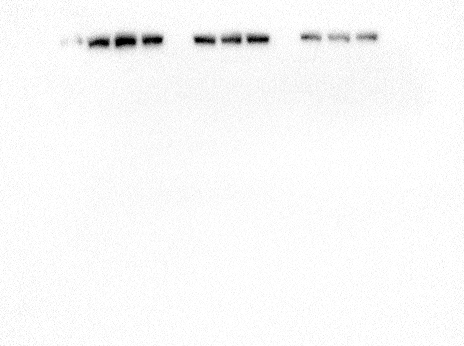

Supplement: Figure 6—source data 2. [file elife-100604-fig6-data2.zip › Fig. 6E (b-catenin).tif]

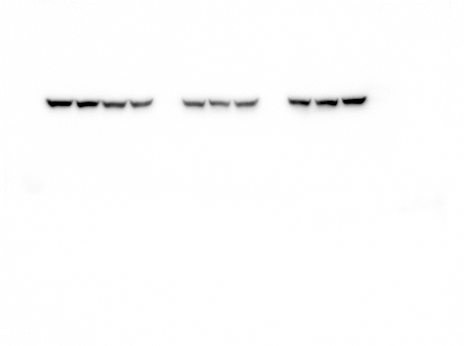

Supplement: Figure 6—source data 2. [file elife-100604-fig6-data2.zip › Fig. 6E(b-actin).tif]
